# Detection of Enteric Viruses and Bacterial Indicators in a Sewage Treatment Center and Shallow Water Bay

**DOI:** 10.3390/ijerph17186483

**Published:** 2020-09-06

**Authors:** Essam M. Janahi, Sakina Mustafa, Saba F. D. Parkar, Humood A. Naser, Zaki M. Eisa

**Affiliations:** 1 Department of Biology, College of Science, University of Bahrain, Sakhir 32038, Bahrain; smustafa@uob.edu.bh (S.M.); sabaparkar@outlook.com (S.F.D.P.); hnaser@uob.edu.bh (H.A.N.); 2 The National Center for Disease Prevention and Control, Jazan 82722-2476, Saudi Arabia; zomar@moh.gov.sa

**Keywords:** enteric viruses, wastewater, PCR, sewage pollution, bacterial indicators

## Abstract

The incidence of enteric viruses in treated wastewater and their potential release into the environment or use for agriculture are very critical matters in public health. In our study, PCR (polymerase chain reaction) analysis of enteric viruses was performed on 59 samples of influents and effluents collected from Tubli wastewater treatment plant (Water Pollution Control Center (WPCC)) and Tubli Bay, where the effluents were discharged, in Kingdom of Bahrain during two sampling periods. Four clinically essential waterborne enteric viruses were examined: enterovirus (EV), hepatitis A virus (HAV), astroviruses (AV), and rotaviruses (RV) and compared to standard bacterial and bacteriophages indicators of fecal pollution. Detection rates of EV, AV, HAV, and RV in the influent samples were 100%, 75%, 12.5%, and 12.5%, respectively, while 50% of the effluent samples from Tubli WPCC contained only EV RNA. None of the tested enteric viruses could be detected in any of the samples collected directly from Tubli Bay. Effluent samples from Tubli plant did not show significant seasonal differences. Since detection of enteric viruses genome does not necessarily indicate infectivity, the infectivity of these viruses was evaluated through isolation and growth of indictor bacteria and bacteriophages. High concentration of fecal bacteriological indicators was detected in all effluents samples (100%): 3.20 × 10^3^ cfu/mL for *E. coli*, 1.32 × 10^3^ cfu/mL for *Salmonella* spp., and 1.92 × 10^3^ cfu/mL for *Shigella* spp. *E. coli* and *Salmonella* specific bacteriophages were also detected in the effluent samples in high titers. The combined results of PCR and bacterial enumeration point to a probable public health risk via the use of these wastewaters in agriculture or their discharge into the sea. Continuous surveillance of viral and bacterial prevalence and their resistance to sewage disinfection procedures could contribute to a better control of risks associated with the recycling of effluent wastewater and its release into the environment.

## 1. Introduction

Despite the decline of waterborne diseases’ incidence over the past decades, due to enhanced water treatment procedures, waterborne epidemics yet persist to happen [1]. Leakage of wastewater and discharge of raw or inadequately treated sewage to the aquatic environment is the main cause of waterborne outbreaks [2,3,4,5]. Microbes found in contaminated water include bacteria, parasites, protozoa, fungi, and viruses [1,6]. About 30–90% of waterborne disease outbreaks are reportedly caused by enteric viruses, making them one of the most common illnesses around the world [7,8,9]. Enteric viruses, which are expelled in human feces, have been detected in rivers, recreational waters, seawater, groundwater, and even sewage treated water [7]. Commonly known human enteric viruses include polioviruses, enteroviruses (EV), coxackieviruses, echoviruses, hepatitis A virus (HAV), noroviruses, sapoviruses, astroviruses (AV), rotaviruses (RV), and adenoviruses [3,7]. Among the ailments caused by them are respiratory infections, conjunctivitis, gastroenteritis, hepatitis, in addition to some more serious diseases including aseptic meningitis, encephalitis, paralysis, and myocarditis [3,4,7]. Although enteric viruses cannot reproduce in water, they are capable of surviving for a long time, up to 130 days, and they are infectious at low doses (1–50 tissue culture infectious units) when ingested [1,4,10,11].

Microbial water quality is currently assessed using bacterial indicators, such as enterococci, total and fecal coliforms [5,12,13,14,15]. This monitoring tool is considered inadequate as there is no correlation between bacterial indicators and other pathogens like parasites and viruses and various studies have detected enteric viruses in water meeting the World Health Organization (WHO) bacterial standards [16,17]. Besides, bacteria are more sensitive to the different sewage treatment processes compared to viruses [6,18,19]. Several factors such as the virus capsid, size, and aggregation with the solid waste materials in sewage can help them survive the exposure to such treatment processes and remain infectious [12,13,18,20]. Most of the viruses (50–90%) can be eliminated during sewage treatment; however, a considerable load of viruses remains infectious and discharged to the aquatic environment, creating possible risk to the public health [1,11].

Kingdom of Bahrain is a small archipelago of more than 33 islands with very dense (2239 inhabitants per km^2^) and rapidly growing population [21]. Bahrain is characterized by low rainfall and shortage of natural water sources, which increased the demand for alternative water sources, like desalinated water and sewage treated effluent. Tubli Water Pollution Control Center (Tubli WPCC) is the main and largest wastewater treatment plant in Bahrain, which receives more than 300,000 m^3^ day^−1^ of wastewater. Since its operation in the early eights, this plant was expanded several times. At Tubli WPCC, the municipal wastewater is subjected to primary, secondary, and tertiary treatments. The treatment of sewage starts by receiving the sewage flow from the collection network in the inlet area. The sewage then goes through screening and is sent to the aeration tanks where air is pumped to the flow. Subsequently, the flow is sent to the clarifiers and then subjected to chlorination. Finally, the flow is sent to the ozone plant for final treatment [21]. Tubli WPCC is currently producing about 150 million liters per day of secondary-treated effluent. Out of this amount, only one third is processed through tertiary treatment, and about half of which is re-used for irrigation and agricultural purposes, while the remaining is dumped into Tubli Bay [22]. Excessive and continuous discharge of sewage effluents in Tubli Bay, a shallow semi-enclosed bay located south of Manama, causes severe pollution and degradation of the marine environment in the bay and nearby regions [23]. Additionally, coastal reclamation intensified along the coastline of Tubli Bay during the last decades, resulting in densely populated residential areas nearby the outfall of Tubli WPPC. This could pose a possible health risk for the public from viruses and microbial pathogens associated with sewage discharge.

The presence of various pathogens in the tertiary treated effluent of wastewater treatment plants has been well documented [24,25,26]; therefore, monitoring pathogens, including viruses, is of high importance for the public health. The main objective of this study was to detect several human enteric viruses (EV, AV, RV, and HAV) in tertiary treated wastewater from Tubli WPCC as well as analyzing water samples from Tubli Bay during two sampling periods, to examine the infectious potential of treated effluents and to estimate the efficiency of decontamination procedures. The PCR test cannot differentiate between infectious and noninfectious virions, so the detection of viral genomes does not certainly show a real risk to public health. Hence, the infectivity of enteric viruses in treated effluents was assessed through the detection and growth of indictor bacteria and bacteriophages, an approach utilizing the precise PCR test with bacterial infectivity assay. Furthermore, it is important to assess if bacterial or bacteriophage indicators are a good choice for detection and viability of enteric viruses. Finally, this study represents the first baseline documentation in Bahrain for the effectiveness of the wastewater treatment processes that can be valuable for environmental risk assessment of sewage plants.

## 2. Materials and Methods

### 2.1. Sample Collection

**Figure 1:**
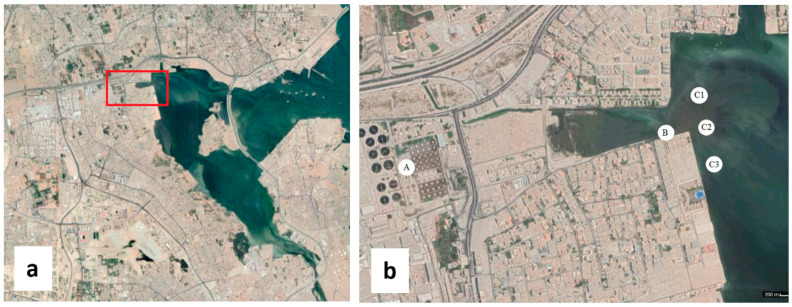
(**a**) Tubli Bay and the location of the sampling area. (**b**) Sampling points A: Influent samples from Tubli Water Pollution Control Center (WPCC), B: Effluent samples from the discharge pipe, C1, C2, and C3: Samples from the bay near the discharge area at different directions.

A total of 59 samples were collected over one-year period during different seasons. Sixteen samples were collected from Tubli WPCC including raw sewage (influent) and tertiary treated sewage (effluent). The rest of the samples were collected from Tubli Bay downstream of the effluent discharge pipe from three different locations (Figure 1). The sample collection was carried out first during winter (January–February) and early summer (May–June). The geographical coordinates of the sampling area, located at the northeast of Tubli Bay, are 26°11′46″ N 50°33′49″ E. The samples were collected in sterile bottles, stored at 4 °C during transport and processed within 24 h.

### 2.2. Virus Concentration

Virus concentration was carried out following the protocol of Katayama et al. [27]. Two liters of each sample were filtered through Whatman filter papers to remove large debris. The samples were then filtered through HA negatively charged filter membranes (0.45 µm pore size) (Merck Millipore, Darmstadt, Germany) to collect the viruses on the membrane. The filters were washed with 200 mL of 0.5 mM H_2_SO_4_ to rinse the positively charged ions, then the viruses were eluted with 10 mL of 1 mM NaOH and recovered with 50 µL of 50 mM H_2_SO_4_ and 50 µL of 100× Tris EDTA (TE) buffer. The samples were stored at −20 °C until further processing. The virus-containing elute (10 mL) was further concentrated using Centriprep Centrifugal Filter Concentrators (Merck Millipore, Darmstadt, Germany) by centrifuging at 1500× *g* for 10 min at 4 °C. The concentrates were rinsed twice with 10 mL RNase free water and a final volume of 2 mL was obtained.

### 2.3. Viral RNA Extraction and Synthesis of Complementary DNA

Viral RNA was extracted using Pure Link Viral DNA/RNA mini kit (Life Technologies, Carlsbad, CA, USA) starting from 200 µL of the concentrated samples. Contaminating DNA was removed using DNase I (Life Technologies, Carlsbad, CA, USA). The manufacturer’s protocol was followed. Five microliters of DNase I buffer and 1.5 μL DNase I were added to the contaminated RNA sample, mixed gently, and incubated at room temperature for 15 min. The DNase was inactivated by adding 1.5 µL of 25 mM EDTA and heating at 65 °C. The complementary DNA (cDNA) of the viral RNA was synthesized by addition of 3 µL of random hexamer, 22.5 µL of viral RNA, 3 µL of dNTP mix (10 mM each) and 7.5 µL of water. The mixture was heated at 56 °C for 5 min, followed by quick chill on ice. Then, 12 µL of 5× first strand buffer, 6 µL of 0.1 M DTT, and 3 µL of RNaseOUT (40 U/µL, Life Technologies, Carlsbad, CA, USA) were added and incubated at 25 °C for 2 min. Finally, 3 µL of Superscript II reverse transcriptase (20U, Life Technologies, Carlsbad, CA, USA) was added and incubated consecutively at 25 °C for 10 min, at 40 °C for 50 min and at 70 °C for 15 min.

### 2.4. Detection of Enteric Viruses by PCR

Five microliters of the cDNA were used for PCR, by mixing with 1.5 mM of MgCl_2_, 0.2 mM of each of the dNTPs, 0.5 µM of each of the forward and reverse primers (Table 1), and 1 unit of Taq polymerase (Life Technologies, Carlsbad, CA, USA). The PCR conditions for each virus are shown in Table 2. To prevent false-positives due to contamination from previous amplification rounds, at least one negative control in each set of amplification was included. The PCR products were analyzed by ethidium bromide staining gel electrophoresis (90 V, 150 mA).

### 2.5. Microbial Indicators

For determination of bacterial indicators (*E. coli*, *Salmonella* spp., and *Shigella* spp.), each sample (5–20 mL) was assayed by cultivation on different selective culture media (nutrient agar, xylose lysine deoxycholate (XLD) agar medium, eosin methylene blue (EMB) agar medium) in accordance with the standard procedures for the inspection of water and wastewater. The plates were incubated for 24–48 h. Bacteriophages were isolated from the sewage samples by double agar layer method. Tryptone soy soft agar (0.7%) containing 100 µL of the sample and 100 µL of the host bacterial cells were poured on the hard nutrient agar plates. Different host bacteria were cultivated with samples containing phages: *E. coli*, *Salmonella* spp., and *Shigella* spp. The plates were incubated at 37 °C and phage plaques were observed after 3–5 h.

### 2.6. Statistical Analysis

Statistical analyses were performed using IBM SPSS Statistics 23 software (IBM, New York, NY, USA). Descriptive statistics (N, % and mean ± standard deviation (SD) were calculated to assess the number of positive samples. The *t*-test was applied to test the difference between detection rates during summer and winter seasons.

## 3. Results

### 3.1. Enteric Viruses Detection

**Figure 2:**
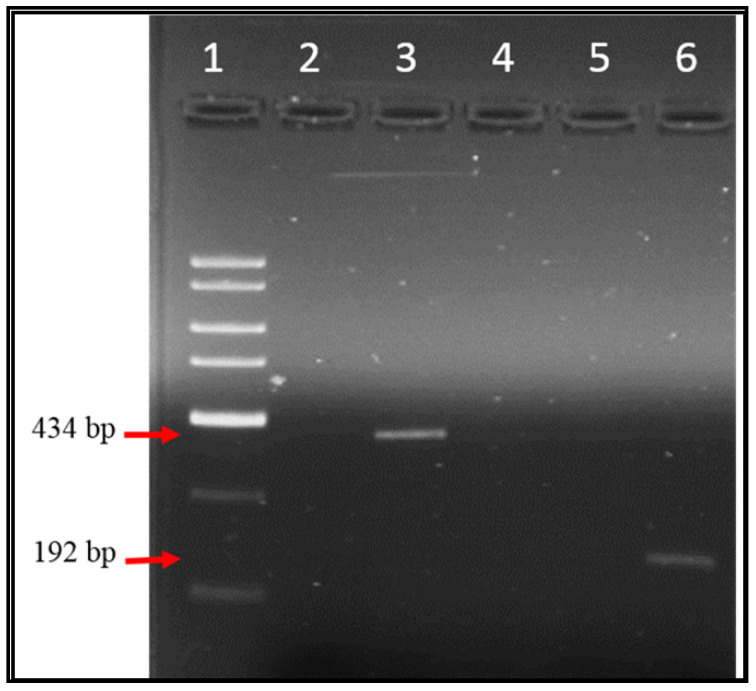
Agarose gel electrophoresis of enterovirus and hepatitis A virus (HAV) RT-PCR products from Tubli WPCC effluent’s sewage samples. Lane 1: 2 Kb DNA ladder, Lane 2: negative control of enterovirus (EV), Lane 3: EV amplicon (434 bp), Lane 5: negative control of HAV, Lane 6: HAV amplicon (192 bp).

**Figure 3:**
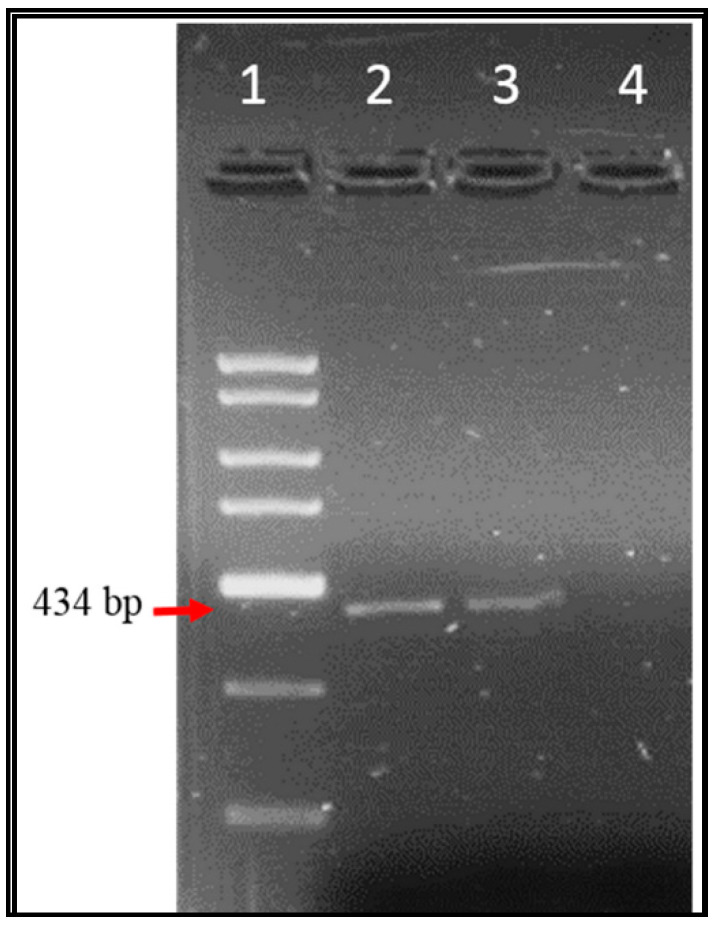
Agarose gel electrophoresis of enterovirus RT-PCR products from Tubli WPCC influent and effluent sewage samples. Lane 1: 2 Kb DNA ladder, lane 2: EV amplicon (434 bp) from influent sewage sample, lane 3: EV amplicon (434 bp) from effluent sewage sample, lane 4: negative control of EV.

**Figure 4:**
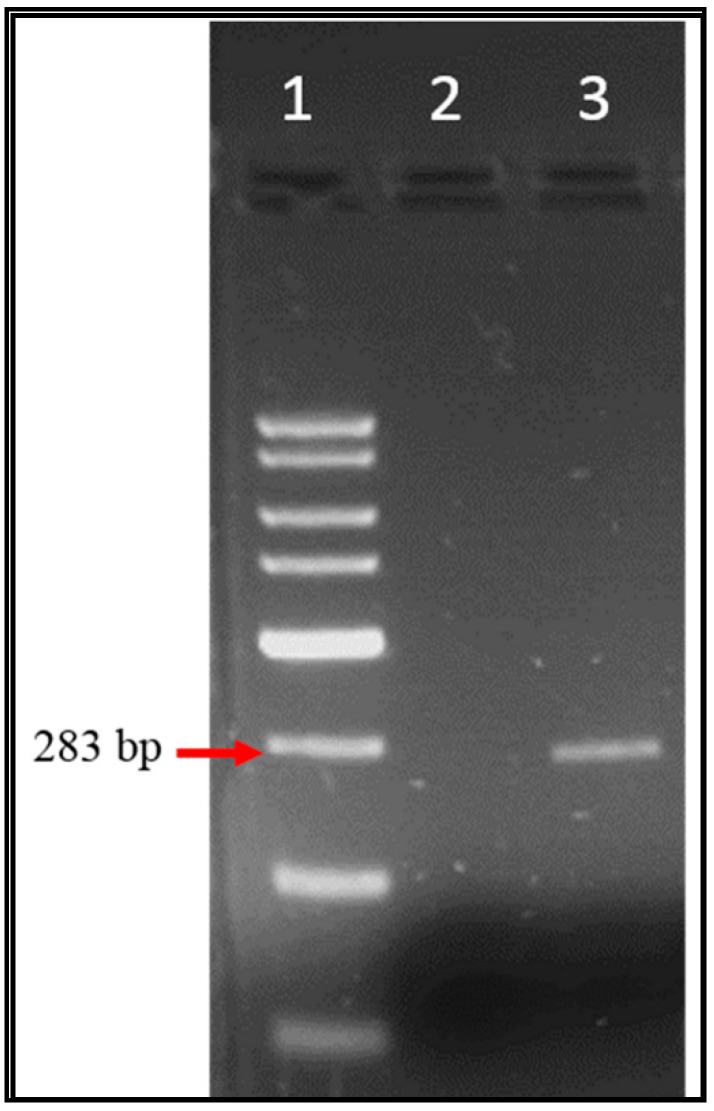
Agarose gel electrophoresis of astrovirus RT_PCR products from Tubli WPCC effluent’s sewage samples. Lane 1: 2 Kb DNA ladder, lane 2: negative control of astroviruses (AV), lane 3: AV amplicon (283 bp).

Over one-year period, 59 samples were examined for EV, AV, HAV, and RV genomes by specific conventional PCR and the amplified products were examined through agarose gel electrophoresis. Figure 2, Figure 3 and Figure 4 illustrate the bands of the PCR amplicons for EV, HAV, and RV. EV had an amplicon of 434 bp in size, AV had an amplicon of 283 bp in size, HAV had an amplicon of 192 bp in size, and RV had an amplicon of 392 bp in size.

**Figure 5:**
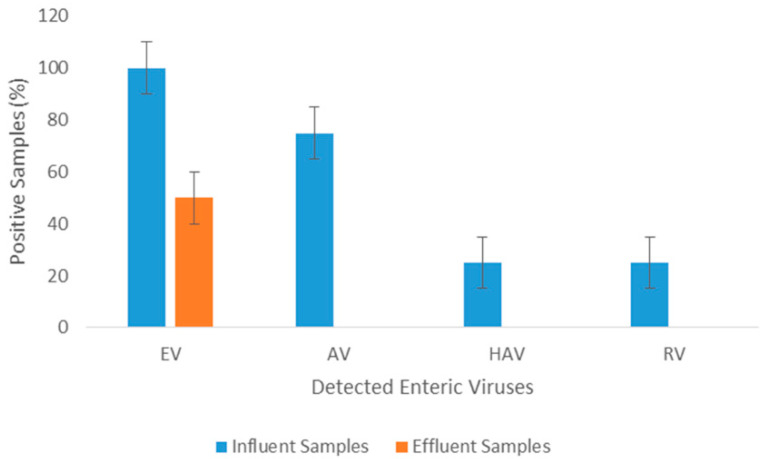
Detection frequencies (%) of enteric viruses (EV, AV, HAV, and rotaviruses (RV)) in the influent and effluent sewage samples during sampling periods.

**Figure 6:**
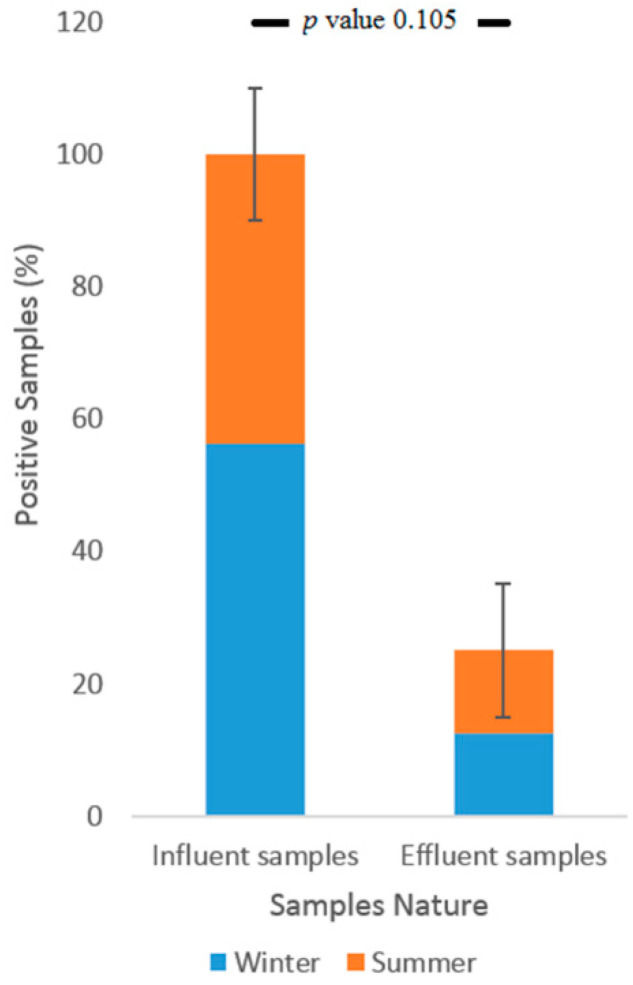
Comparisons of detection frequencies (%) of enteric viruses during summer and winter seasons.

All of the Tubli WPCC influent samples contained EV RNA, whereas the detection rate of the effluent samples (undergone through all treatment processes) was only 50% (Figure 5). AV, HAV, and RV were detected in 75%, 12.5%, and 12.5%, respectively, of the influent sewage samples, while effluent samples showed no traces of them. Tertiary treatment, after chlorination, displayed removal efficiencies of about 50% of EV. None of the tested enteric viruses could be detected in any of the samples collected directly from Tubli Bay (data not shown). Effluent samples from Tubli WPCC did not show significant variation during the different seasonal sampling periods as shown in Figure 6.

### 3.2. Microbiological Indicators

To confirm the viability of the detected enteric viruses, the effluent samples were analyzed for different fecal coliforms and their specific phages. Enumeration of *Salmonella* spp., *E. coli*, and *Shigella* spp. was carried out according to the Most Probable Number (MPN) method and cultivated on different selective media. The mean concentration of fecal bacteriological indicators in effluent samples were 3.20 × 10^3^ cfu/mL for *E. coli*, 1.32 × 10^3^ cfu/100 mL for *Salmonella* spp., and 1.92 × 10^3^ cfu/mL for *Shigella* spp. (Table 3). The mean count of *E. coli* was higher than that of *Salmonella* spp. and *Shigella* spp. *E. coli* and *Salmonella* spp. specific bacteriophages were detected and enumerated from the effluent samples according to standard procedures (Table 3). *E. coli* phages were the most abundant in the samples having values ranging between 7 and 8 log pfu/mL, while *Salmonella* spp. specific phages having lower values ranging between 3 and 4 log pfu/mL (Table 3). In raw sewage, mean concentrations of bacteriophages indicators were ranging between 5.6 × 10^5^ and 8.2 × 10^10^ pfu/mL. The effluent of Tubli WPCC displayed a clear decline in the concentration of *E. coli* bacteriophages from 8.2 × 10^10^ to 2.15 × 10^8^ pfu/mL, which corresponds to a reduction of more than 99% by the treatment procedures.

## 4. Discussion

Several studies showed high levels of enteric viruses in wastewaters even after decontamination treatment [30,31,32,33]. Kokkinos et al. detected EV in 12% of the effluent samples and showed negative results for HAV from a primary wastewater treatment plant in Greece [34]. El-Senousy et al. have detected AV in 23% of the raw sewage samples and in 2% of the effluent samples obtained from three sewage treatment plants in Egypt [35]. Hence, sewage wastewater should be processed properly before discharge to the environment, as low doses of viruses, up to 50 infectious units, pose a serious health risk to the public [11]. In the course of this investigation, sensitive PCR assays were used to identify EV, AV, RV, and HAV genomes from effluent samples of Tubli WPCC during different sampling seasons. The enterovirus was the only enteric virus detected in the analyzed samples and it was detected in half of the samples. Some viruses can resist the different treatment processes, especially when there are high organic particles in the water, as it protects viruses from disinfection [10]. None of the other tested enteric viruses were detected, which implies that the only detected enterovirus seems to be more genetically resistant than other enteric viruses and thus tolerate the disinfection processes. This result is of significant importance since tertiary treated wastewater is used for agricultural irrigations. Enterovirus’s stability depends on temperature, UV radiation, and humidity in the external environment. It is stable for 1–3 h at pH levels of 3–5. It resists proteolytic enzymes, bile salts, and high concentrations of sodium chloride. Therefore, it can survive in water environments for a long time [36].

Sometimes seasonal circulation of enteric viruses can result in variable detection rates. For example, seasonal peaks for RV were noticed in the treated wastewater during first and fourth annual quarters [37]. However, our data show no statistical differences (*p* value = 0.105) in the detection rate of enteric viruses in summer as compared with winter. In a study by Tani et al., the peak level of enterovirus was observed during the summer season; however, it was still detected during the autumn and winter [38]. So, there could be seasonal variation in our samples in terms of quantity.

Coastal water and beaches that receive sewage wastewater put swimmers and divers at higher risk of contracting eye, ear, gastrointestinal, or respiratory viral infections [10]. In addition, edible shellfishes in contaminated waters pose high risk for the public. Shellfishes are filter feeders and hence accumulate viruses and other microbes in high concentrations in their tissues [4]. EV was detected in 55% of beach samples in Spain by Mocé-Llivina et al. [39]. RV was also detected by qPCR from the Buffalo River in the Eastern Cape Province of South Africa by Chigor et al. [7]. The samples from Tubli Bay did not show any positive indications of the presence of enteric viruses. Tubli Bay is where most of the secondary and tertiary-treated sewage effluents are disposed, so it was expected to show considerable amount of different enteric viruses. Several factors can affect virus concentration and detection and hence the viral load in these samples. Our negative results might be due to the dilution effects in the mixing zone of the discharge area, which results in the removal of viruses from the tested water through flocculation and sedimentation procedures. Furthermore, environmental factors like temperature and ultraviolet radiation can affect the stability of these viruses in seawater by damaging the viral nucleic acid and proteins. In addition, water salinity, which is quite high in the region, can cause viral aggregation and hence lower viral titers [40]. Furthermore, the study area is characterized by high organic matter pollution from the continuous disposal of inadequately treated sewage, so it is highly probable that high concentrations of extracellular proteases, nucleases, and other enzymes could damage the virus capsid and result in genome degradation [23,40]. Our negative results could also be a consequence of the presence of high concentrations of PCR or RT inhibitory substances in seawater [12]. Highly polluted seawater with municipal waste is full of humic compounds, divalent cations, salts, and other inhibitory substances, which could affect the PCR efficiency and give false-negative results [18]. Kopecka et al. suggested that the virus concentration steps lead to enrichment of impurities as well [41].

The PCR method cannot differentiate between infectious and noninfectious virions, so the detection of viral genomes does not positively show their infectivity, which is the real risk to public health. Hence, the infectivity of enteric viruses in treated effluents was assessed through the detection and growth of indictor bacteria and bacteriophages. All tested effluent samples showed high counts of *E. coli*, *Salmonella* spp., and *Shigella* spp. According to WHO guidelines for the microbiological quality of treated wastewater used in agriculture, the fecal coliform counts for unrestricted irrigation water should be less than 10 bacteria/mL [42]. In our study, *E. coli* and other coliforms counts were much higher, as their population exceeded 10^3^ bacteria/mL. Therefore, our study suggests a positive correlation between the viability of enteric viruses and viability of indicator bacteria. Such positive correlation was reported in several studies [24,43,44].

A critical finding of our study is the detection of *Salmonella* spp. in the effluent samples, which is associated mainly with gastroenteritis but may develop life-threatening complications [45]. Primary, secondary, and tertiary wastewater treatments are expected to eliminate 2 to 6 log units of the enteric microorganisms [46]. However, due to large quantity of bacteria in raw sewage, nonresponsive operations, or inadequate plant maintenance, pathogens can still be detected after final treatment [47]. This serious public health risk can be worsened by the ability of *Salmonella* to survive in the environment for long times, particularly in high salty conditions, and its ability to mutate and develop antibiotic resistance [48,49]. *Salmonella* isolated from effluent had been shown to have higher antibiotic resistance rates compared with those of influent [48].

Most regulations and guidelines for reusing treated wastewater for irrigation utilizes total coliforms, fecal coliforms, or *E. coli* as indicator microorganisms to monitor the bacterial quality [50]. However, there is no universal agreement regarding these guidelines between different countries and organizations. For example, the following countries have different guidelines for the microbial quality of irrigation water (cfu/100 mL): Italy (*E. coli* ≤ 10), Jordan (*E. coli* or Fecal Coliform ≤ 100), Kuwait (Total Coliforms ≤ 400), Saudi Arabia (Thermo-Tolerant Coliform ≤ 1000), and Bahrain (Total Coliforms ≤ 1000) [22,50]. In a study by Stine et al., they estimated *Salmonella* concentrations range that would result in a 10−4 annual risk of infection from using treated wastewater between 1.5 × 10^2^ cfu/100 mL and 7.2 × 10^6^ cfu/100 mL [51]. Some researchers pointed out that some of the current regulations and guidelines are not sufficient to ensure the safety of reusing the treated wastewater for agriculture. For instance in Bolivia, it has been reported that using treated wastewater that meets WHO guidelines has resulted in 37% of Bolivia’s overall diarrheal disease prevalence [52].

Since bacterial indicators are not always correlated with virus contamination of water, as they are more easily inactivated by wastewater decontamination procedures, enteric viruses have been suggested as a possible indicator for fecal contamination of water [13]. However, their detection, quantification, and viability tests are cumbersome, time-consuming, and quite expensive [11]. Hence, bacteriophages provide a good alternative as indicators for water microbial quality especially by considering that they are highly resistant against environmental factors [1,18]. All our enteric virus-positive samples had high titers of bacteriophages of 5–10 log pfu/mL in the influent and 3–8 log pfu/mL in the effluent. Despite that the bacteriophage load declined by 1–2 log pfu/mL units in influent and effluent samples, it is clear that the utilized wastewater decontamination processes did not decrease the phage titer beyond detection level. Bacteriophages and especially coliphages share some characteristics with human enteric viruses, like size, structure, and morphology. In addition, both enteric viruses and male-specific coliphages, in particular, can replicate in the gastrointestinal tracts of humans. Nieuwstad et al. proposed the use of somatic coliphages as indicators for fecal contamination [53]. Detection of the non-pathogenic coliphages is much simpler and rapid, making them a good indicator of water quality measurements [7,14]. Despite the apparent decline in the coliphages concentration as a result of the treatment procedures in Tubli WPCC, still some infectious enteric viruses and bacteria were detected at levels above the acceptable standards of WHO.

## 5. Conclusions

In conclusion, enterovirus was detected in tertiary treated sewage water from Tubli WPCC, which is used for irrigation purposes, suggesting it could be resistant to the currently applied treatment processes in Tubli WPCC. There was no seasonal variation in the detection rates. Bacterial pathogens were also detected at high concentrations in effluent samples, such as *E. coli*, *Salmonella* spp., and *Shigella* spp. There was also a positive correlation between the detection of EV and detection and cultivation of pathogenic bacteria and their specific bacteriophages, which implies that the detected EV was viable and infectious. Furthermore, no enteric viruses were detected in the collected samples from Tubli Bay, though this does not rule out their presence due to the aforementioned factors. The resistance of microbes to sewage treatment processes and prolonged survival in water environments may possess a possible risk to the public health. Because of the concern for public health, routine surveillance for the treated effluents as well as Tubli Bay should be carried out regularly to screen for enteric viruses. Our findings highlight the need for new and more thorough national guidelines for the use of treated effluent in irrigation especially in terms of viral and bacterial monitoring. Reports from monitoring enteric viruses in the USA showed sudden mutations can change the circulation pattern and may lead to large-scale epidemics [54]. The current disinfection protocols in Tubli WPCC would benefit from reviewing and potential improvement. Further research is recommended to quantify enteric viruses via qPCR and also detect enteric viruses in shellfish of Tubli Bay, especially by considering that some expats harvest them from the area and shellfish are well-known risk factors for gastroenteritis and hepatitis.

## Figures and Tables

**Table 1:** PCR primer sequences used for detection of the different enteric viruses.

Virus	Primer Sequence (5′→3′)	Amplicon Size (bp)	Map Position	Reference
**EV**	FW: 5′-CAAGCACTTCTGTTTCCCCGG-3′ RV: 5′-ATTGTCACCATAAGCAGCCA-3′	434	162–182 577–596	[28]
	FW: 5′-TCCGGCCCCTGAATGCGG-3′ RV: 5′-CACCGGATGGCCAATCCAAT-3′	196	446–463 623–642	[28]
**AV**	FW: 5′-CGTCATTATTTGTTGTCATACT-3′ RV: 5′-ACATGTGCTGCTGTTACTATG-3′	289	1182–1203 1450–1470	[29]
**HAV**	FW: 5′-CAGCACATCAGAAAGGTGAG-3′ RV: 5′-CTCCAGAATCATCTCCAAC-3′	192	2035–2054 2208–2226	[27]
**RV**	FW:5′-GGCTTTAAAAGAGAGAATTTCCGTCTGG-3′ RV: 5′-GATCCTGTTGGCCATCC-3′	392	1–28 376–392	[28]

**Table 2:** PCR cycling conditions.

Virus	PCR Conditions (Temperature and Time)					
	Cycles	Initial Denaturation	Template Denaturation	Primer Annealing	Primer Extension	Final Extension
**EV**	35	94 °C, 3 min	94 °C, 45 s	55 °C, 30 s	72 °C, 60 s	72 °C, 10 min
**AV**	30	94 °C, 3 min	94 °C, 30 s	50 °C, 20 s	72 °C, 30 s	72 °C, 5 min
**HAV**	35	94 °C, 3 min	94 °C, 1 min	51 °C, 1:30 min	72 °C, 1 min	72 °C, 7 min
**RV**	40	94 °C, 3 min	94 °C, 30 s	50 °C, 20 s	72 °C, 30 s	72 °C, 5 min

**Table 3:** Detection and quantification of indicator bacteria and their specific phages from Tubli WPCC wastewater.

Detected Bacteria	Mean Total Count of Bacteria (cfu/mL) ^1^	Mean Titer of Bacteria-Specific Phages (pfu/mL) ^2^	
		Influent	Effluent
*E. coli*	3.20 × 10^3^	8.2 × 10^10^	2.15 × 10^8^
*Salmonella* spp.	1.32 × 10^3^	5.6 × 10^5^	2.77 × 10^3^
*Shigella* spp.	1.92 × 10^3^	ND *	ND *

* Not Determined, ^1^ colony-forming unit, ^2^ PFU: plaque-forming unit.
